# Receptor guanylyl cyclase-G is a sensory protein activated by cool temperatures and predator odor 2,4,5-trimethylthiazoline

**DOI:** 10.1186/2050-6511-16-S1-A29

**Published:** 2015-09-02

**Authors:** Ruey-Bing Yang

**Affiliations:** 1 Institute of Biomedical Sciences, Academia Sinica, Taipei, Taiwan; 2 Institute of Pharmacology, School of Medicine, National Yang-Ming University, Taipei, Taiwan

## Background

Receptor guanylyl cyclase (GC)-G is highly expressed in neurons of the Grueneberg ganglion (GG) in the anterior region of the murine nose, whose cells are activated by cool temperatures and volatile danger cues such as alarm pheromones and other structurally similar semiochemicals involuntary released by predators [1,2]. The predator odor 2,4,5-trimethylthiazoline (TMT), a volatile scent originally isolated from fox feces, induces robust innate freezing behaviors in rodents [3]. To date, molecular identities of the GG sensors for cool temperatures and pheromones are largely unclear. In addition to GC-G, GG neurons express signaling elements associated with cyclic guanosine monophosphate (cGMP), including the cGMP-stimulated phosphodiesterase 2A and the cGMP-activated ion channel CNGA3 [4-6]. Interestingly, recent reports suggest that cGMP signaling is crucial for thermal transduction and odorant-evoked electrical responses in the GG neurons [6,7]. However, whether GC-G directly acts as a sensor for cool temperatures and/or predator odor TMT remain elusive.

## Materials and methods

A combination of biochemical and molecular biology methods, Ca2+ imaging as well as behavioural studies comparing wild-type and GC-G-knockout (KO) mice was used to elucidate the molecular and biological function of GC-G in sensing cool temperatures and predator scent TMT.

## Results

We show that GC-G is a dual thermosensory and pheromone receptor that can be maximally stimulated by cool temperatures of about 15°C and TMT in both in vivo cellular cGMP accumulation assays and in vitro GC assays. Mechanistically, while coolness enhances dimerization of GC-G, TMT directly binds the extracellular domain of GC-G to stimulate its enzymatic activity. Consistent with these findings, we observed substantially reduced coolness-induced responses of GG neurons and coolness-evoked ultrasonic vocalization in GC-G-KO pups. Likewise, TMT-induced calcium transients in the GG as well as TMT-evoked innate fear behaviors and an increase of serum corticosterone (a stress hormone) were markedly attenuated in the GC-G-KO mice compared to wild-type littermates.

## Conclusions

Our data suggest that GC-G is a double functional receptor in the GG for sensing cool temperatures and predator scents in an age-specific manner. Whereas GC-G activation in the GG by coolness is critical for the generation of ultrasound calls by isolated pups to elicit maternal care [8], GC-G can detect the predator odor TMT to evoke innate defensive responses in adult mice to maximize species survival.
